# Investigating the relationship between cultural intelligence and cultural competence among students in nursing faculties in Iran: an analytical cross-sectional study

**DOI:** 10.1186/s12912-025-03555-2

**Published:** 2025-07-09

**Authors:** Fatemeh Ahmadi Forg, Pardis Doosti, Hadi Hasani, Fatemeh Heydari, Mohammadjavad Roshanfekr, Leila Hassangholizadeh, Fahimeh Ramezani, Solmaz Vahedi

**Affiliations:** 1 Department of Nursing, Tabas School of Nursing, Birjand University of Medical Sciences, Birjand, Iran; 2 Department of Medical-Surgical Nursing, School of Nursing and Midwifery, Tehran University of Medical Sciences, Tehran, Iran; 3 Students’ Scientific Research Center, Tehran University of Medical Sciences, Tehran, Iran; 4 Department of Epidemiology, Faculty of Health, Ilam University of Medical Sciences, Ilam, Iran; 5 Department of Nursing, School of Nursing and Midwifery, Sabzevar University of Medical Sciences, Sabzevar, Iran; 6 Student Research Committee, Mashhad University of Medical Sciences, Mashhad, Iran; 7 Student Research Committee, Hamadan University of Medical Sciences, Hamadan, Iran; 8 Department of Nursing Management, School of Nursing & Midwifery, Tehran University of Medical Sciences, Tehran, Iran

**Keywords:** Cultural intelligence, Cultural competence, Nurse, Nursing students

## Abstract

**Background and objective:**

Given the multicultural and multiethnic environment in Iran, as well as the growth of medical tourism, the development of culturally congruent care is a key necessity for enhancing the quality and safety of nursing care. This study aims to investigate the relationship between cultural intelligence and cultural competence among students in nursing faculties.

**Materials and methods:**

This analytical cross-sectional study was conducted among 378 students in nursing faculties from the Jovein and Tabas Nursing Faculties, who were selected through stratified sampling. Data were collected via standardized questionnaires for cultural intelligence and the Critical Cultural Competence Scale (CCCS). Data analysis was performed via descriptive statistics and multiple regression analysis with SPSS software version 23.

**Findings:**

The participants were nearly equally distributed in terms of sex (54.2% female) and were mostly single (91.8%). The overall mean cultural intelligence score of the participants was 101.66 ± 14.40, and the mean cultural competence score was 100.38 ± 10.89. According to the results of multiple regression analysis, a significant relationship was observed between the cultural intelligence and cultural competence of the students (β = 0.524, *p* < 0.001).

**Discussion and conclusion:**

Based on the findings of this study, which highlight the relationship between cultural intelligence and cultural competence among students in nursing faculties, it seems necessary to focus on enhancing cultural intelligence as a means to improve the cultural competence of nursing students. This, in turn, is crucial for improving the quality of healthcare services.

## Introduction

In today’s increasingly interconnected world, cultural differences are becoming more visible and meaningful, especially in the field of healthcare [1]. Globalization and patterns of migration have introduced new complexities for healthcare systems, particularly those serving multicultural and diverse communities. As a result, many healthcare systems have recognized the importance of offering culturally sensitive care to improve the quality of services and ensure fair, respectful treatment for all patients, regardless of their cultural background [2]. For many patients, cultural differences between their own values and those of healthcare providers can lead to miscommunication, discomfort, or even inadequate care. The growing diversity within patient populations has therefore made culturally congruent or culturally tailored care not only desirable but also necessary [3]. Cultural competence in nursing refers to the ability of healthcare providers to understand, respect, and effectively interact with patients from diverse cultural backgrounds. It includes awareness of one’s own cultural worldview, knowledge of different cultural practices, and cross-cultural skills to deliver patient-centered care [4, 5]. Leininger defines culturally competent care as care that is “culturally congruent,” meaning that it fits with patients’ values, beliefs, and lifeways. She emphasized the importance of understanding cultural variations in health and illness [4, 6].

Iran is a culturally diverse country with various ethnic, linguistic, and religious groups [7]. While Persians form the majority, minorities such as Azeris, Kurds, Lurs, Arabs, Baluchis, and Turkmens maintain distinct cultural identities [8]. Persian is the official language, but several regional languages, such as Azerbaijani, Kurdish, and Arabic, are also spoken [9]. Although most Iranians are Twelver Shi’a Muslims, there are also Sunni Muslims, Christians, Jews, and Zoroastrians with legal recognition [7]. This cultural diversity influences communication styles, health beliefs, and caregiving practices [10]. In a country like Iran, with its rich cultural, ethnic, and linguistic diversity, providing culturally sensitive care and investing in cultural competence training for healthcare professionals has become a vital strategy for enhancing both the quality and safety of nursing care. Moreover, the rapid growth of the medical tourism industry, especially in developing Asian nations such as Iran, is creating new opportunities and challenges. Nurses, nursing students, and faculty members play crucial roles in this emerging sector. It is essential that these healthcare professionals strengthen both their scientific knowledge and cultural competencies to help elevate the country’s medical tourism industry to a competitive international level [11].

When healthcare settings fail to address cultural and linguistic diversity, the consequences can extend far beyond individual patient experiences. Injustices and inequities in health outcomes can emerge, endangering not only the physical but also the mental, social, and spiritual well-being of patients, their families, and the healthcare workforce itself [12]. Cultural diversity is shaped by factors such as traditions, beliefs, ethical values, perceptions of health and illness, and language barriers; when these factors go unrecognized, they can foster misunderstandings, conflicts, ethnocentrism, discrimination, and harmful stereotypes. These challenges are deeply rooted in how people perceive cultural differences and in the unique historical experiences that shape the identities of different cultural groups [13].

Considering both Iran’s diverse cultural landscape and the expansion of its medical tourism industry, there is a clear and pressing need for culturally congruent care to improve nursing services. In addition, the increasing trend of nursing students and professionals seeking further education in developed countries has made it even more important to cultivate cultural competence as a core skill in nursing education. The development of this competency is likely to have a meaningful impact on the global future of nursing practice and education [11].

One valuable concept in this context is cultural intelligence, first introduced by Earley and Ang from the London Business School. They described it as the ability to acquire new cultural knowledge and to respond appropriately to different cultural situations through adapted behaviors [14]. Cultural intelligence is a relatively recent and increasingly important form of intelligence that is particularly relevant in multicultural work environments. According to Peterson (2004), cultural intelligence reflects an individual’s ability to effectively apply their skills and abilities in a variety of cultural contexts. Alarmingly, studies indicate that many professional and business failures occur when people lack a proper understanding of one another’s cultural backgrounds [15]. Thomas and colleagues (2008) expanded on this by defining cultural intelligence as a system of interactive abilities that enable people to comprehend and act effectively in diverse cultural settings [16].

Encouragingly, research has shown that cultural intelligence is not an innate trait but rather a skill that can be developed through formal education and frequent intercultural encounters. This offers organizations and educational institutions a valuable opportunity to build this capability within their teams. In a study conducted by Van Dyne and Ang [17], the various dimensions of cultural intelligence were examined in relation to factors such as adaptability, decision-making, cultural adjustment, and job performance. The findings of this study demonstrated that cultural intelligence plays a predictive role in these areas. For example, the strategy and knowledge components of cultural intelligence were found to influence cultural decision-making, whereas the behavioral and motivational components predicted how well individuals adapted to unfamiliar cultural environments. Additionally, both the strategy and behavioral components have been shown to affect job performance [18]. In light of these insights, the present study aims to investigate the relationship between cultural intelligence and cultural competence among nursing students, a connection that may have significant implications for the future of culturally sensitive nursing education and practice.

## Materials and methods

The present study is an analytical cross-sectional study conducted to examine the relationship between cultural intelligence and cultural competence among students in the nursing faculties of Jovein and Tabas in 2023. A total of 550 students were studying at the universities where the study was conducted. Based on prior research [19], the sample size was determined to be 384 via a standard sampling formula for an unlimited population. However, owing to the voluntary nature of participation, a total of 378 students ultimately participated in the study.

Efforts were made to invite all the students within the study area to participate. Despite our aim for maximum participation, a small number of students either declined to participate or were unavailable during the data collection period. This issue was acknowledged and is not expected to have had a significant effect on the representativeness of the sample. Participants were selected through stratified sampling from the nursing and EMS student populations of these two faculties. After obtaining the ethics code and permission from the Research Vice-Chancellor of Jovein and Sabzevar University of Medical Sciences, the researcher proceeded with sampling and distributed the questionnaires among the students. Before the questionnaires were distributed, the objectives and methods of the study were explained to the students, and their informed consent was obtained. The participants were assured that their names and addresses would not be disclosed and that all their information would be kept confidential. They were also informed that they could withdraw from the study at any time if they wished. The study was approved by the Research Vice-Chancellor of Sabzevar University of Medical Sciences under the ethics code IR.MEDSAB.REC.1402.103, and it was conducted in accordance with ethical standards.

Data collection was conducted using three instruments: a demographic information questionnaire, the Critical Cultural Competence Scale, and the Cultural Intelligence Questionnaire for nurses, designed by Ang et al.

### Demographic information questionnaire

This instrument collected demographic and professional data, including age, gender, marital status, level of education, ethnic and racial background, participation in training programs, and the level of cultural diversity among patients in the hospital where students received training.

### The critical cultural competence scale (CCCS)

Developed and validated by Almutairi et al. (2013), the CCCS assesses healthcare providers’ perceptions of their critical cultural competence in multicultural healthcare environments. The scale includes 43 items distributed across four subscales: critical awareness (12 items), critical knowledge (7 items), critical skills (7 items), and critical empowerment (17 items).

The subscales align with different domains: critical awareness and knowledge with the cognitive domain, critical skills with the behavioral domain, and critical empowerment with the emotional domain. The responses are rated on a 7-point Likert scale ranging from “strongly disagree/never” (1 point) to “strongly agree/always” (7 points). For items assessing the frequency of behaviors, responses range from “never” to “always.” Negatively worded items are reverse-scored. The total score is calculated by averaging item responses, with a score of 5 or above indicating a positive perception of one’s critical cultural competence.

The original scale’s validity and psychometric properties were established by its developers. Content validity was evaluated by subject matter experts and faculty members, whereas construct validity was confirmed through convergent and discriminant validity analyses. The scale demonstrated good internal consistency reliability, with a Cronbach’s alpha of 0.86 for the total scale and subscale values of 0.60 for critical awareness, 0.70 for critical knowledge, 0.77 for critical skills, and 0.86 for critical empowerment [12, 13, 20]. The Cronbach’s alpha was reported as 0.85 for the Clinical Cultural Competence Questionnaire.

### Cultural intelligence questionnaire

This questionnaire, designed by Ang et al. (2007) [21], consists of 20 items grouped into four subscales. Items 1–4 assess the metacognitive (strategic) dimension, items 5–10 evaluate the cognitive (knowledge-based) dimension, items 11–15 measure the motivational dimension, and items 16–20 assess the behavioral dimension. Responses are recorded on a 7-point scale ranging from “strongly disagree” (1 point) to “strongly agree” (7 points). Scores are summed to reflect an individual’s ability to function effectively in culturally diverse situations. The overall score ranges from 20 to 140, with subscales ranging from 4 to 28 for the metacognitive, 6 to 42 for the cognitive, and 5 to 35 each for the motivational and behavioral dimensions. The face and content validity of this questionnaire were confirmed by psychometric experts. In this study, the Cronbach’s alpha coefficient was reported as 0.75, indicating acceptable reliability [21]. The Cronbach’s alpha was reported as 0.91 for the Cultural Intelligence Questionnaire in this study.

Following the approval of the study by the Research Vice-Chancellor of Jovein and Tabas University of Medical Sciences and the acquisition of the relevant ethics code, the researcher initiated the sampling process and distributed the questionnaires among the students. Before participation, the study’s objectives and procedures were explained to the students, and informed consent was obtained from all participants. They were assured of the confidentiality of their personal information, with names and contact details remaining undisclosed. The participants were also informed of their right to withdraw from the study at any time without any consequences [22].

For data analysis, SPSS software version 23 was used. Qualitative variables are presented as frequencies and percentages, whereas quantitative variables are presented as the means and standard deviations. The Shapiro‒Wilk test was used to assess the normality of the quantitative data, and multiple regression analysis was applied to examine the relationships between cultural competence and associated factors. For inferential analysis, multiple linear regression modeling was employed to identify the predictors of cultural intelligence and cultural competence. A significance level of 0.05 was used for all the statistical tests.

## Results

Based on the statistical sample size formula, a sample of 384 participants was initially estimated; however, due to incomplete questionnaires, 378 participants were ultimately included in the analysis. The minimum age of the participants was 18 years. Among the total sample, 54.2% were female, and 91.8% were single.

### Cultural intelligence

As shown in Table 1, the lowest mean score among the domains of cultural intelligence was observed in the metacognitive dimension (mean = 21.25, SD = 3.22), whereas the cognitive dimension had the highest mean score (mean = 27.43, SD = 6.39). The motivational and behavioral dimensions had relatively similar mean values of 26.42 (SD = 4.94) and 26.55 (SD = 4.23), respectively. The overall mean score for cultural intelligence was 101.67 (SD = 14.40), with scores ranging from 64 to 140.

**Table 1:** Mean cultural intelligence scores of students in nursing faculties at Jovein and Tabas faculties

Dimensions of cultural intelligence	Mean	Standard deviation	Maximum	Minimum
Metacognitive	21.25	3.22	28	11
Cognitive	27.43	6.39	41	6
Motivational	26.42	4.94	35	9
Behavioral	26.55	4.23	35	13
Total	101.66	14.40	139	64

### Cultural competence

Table 2 presents the descriptive statistics for the dimensions of cultural competence. The cultural desire domain had the lowest mean score (mean = 11.66, SD = 1.79), whereas the highest score was observed for flexibility (mean = 23.27, SD = 3.74). Other dimensions, such as cultural care (mean = 16.24, SD = 2.67), cultural skills (mean = 17.17, SD = 2.12), cultural knowledge (mean = 15.20, SD = 2.06), and cultural assessment (mean = 16.82, SD = 2.47), fell within a moderate range. The total mean score for cultural competence was 100.38 (SD = 10.89), with observed scores ranging from 59 to 123.

**Table 2:** Mean cultural competence scores of students in nursing faculties at Jovein and Tabas faculties

Dimensions of cultural competence	Mean	Standard deviation	Maximum	Minimum
Flexibility	23.27	3.74	30	14
Cultural Skills	17.17	2.12	22	11
Cultural Knowledge	15.20	2.06	19	10
Cultural Desire	11.66	1.79	15	6
Cultural Care	16.24	2.67	20	7
Cultural Assessment	16.82	2.47	20	9
Total	100.38	10.89	123	59

### Regression analyses

The assumptions of multiple regression were tested and met for both dependent variables: cultural intelligence and cultural competence. The R² value was reported as 0.31 for the cultural intelligence model (Table 3) and 0.39 for the cultural competence model (Table 4).

### Predictors of cultural intelligence

As presented in Table 3, the results of the multiple regression analysis indicated that age (β = 0.246, *p* = 0.005), gender (β = 0.179, *p* = 0.003), and marital status (β = − 0.200, *p* = 0.003) were significant predictors of cultural intelligence. The field of study also showed a significant inverse relationship (β = − 0.250, *p* = 0.001). The academic term was not a significant predictor (*p* = 0.087).

**Table 3:** Results of the multiple regression model for cultural intelligence (adjusted effects: adjusted for age, field of study, gender, marital status, and academic semester)

Variable	Standardized coefficient	Significance (p-value)	Tolerance	VIF	95% confidence interval	
					Lower Bound	Upper Bound
Age	0.246	0.005	0.423	2.366	0.425	315/2
Field of Study	-0.250	0.001	0.597	1.674	-18.017	913/4-
Gender	0.179	0.003	0.849	1.178	1.905	569/9
Marital Status	-0.200	0.003	0.707	1.414	-17.058	550/3
Academic Semester	0.120	0.087	0.644	1.553	0.147	162/2
R²=0.31						

### Predictors of cultural competence

According to Table 4, cultural intelligence demonstrated the strongest positive association with cultural competence (β = 0.524, *p* < 0.001). Other significant predictors included gender (β = 0.164, *p* = 0.003), marital status (β = 0.165, *p* = 0.006), and field of study (β = 0.154, *p* = 0.019). In contrast, age and academic term were not found to be significant contributors (*p* > 0.05).

**Table 4:** Results of the multiple regression model for cultural competence (adjusted effects: adjusted for age, field of study, gender, marital status, academic semester, and cultural intelligence)

Variable	Standardized coefficient	Significance (*p*-value)	Tolerance	VIF	95% confidence interval	
					Lower Bound	Upper Bound
Age	-0.073	0.341	0.411	2.434	-0.861	299/0
Field of Study	0.154	0.019	0.573	1.744	0.813	904/8
Gender	0.164	0.003	0.824	1.214	1.249	956/5
Marital Status	0.165	0.006	0.686	1.459	1.709	011/10
Academic Semester	-0.042	0.496	0.637	1.569	-0.945	459/0
Cultural Intelligence	0.524	< 0.001	0.894	1.118	0.289	430/0
R²=0.31						

## Discussion

Based on the findings of this study, cultural competence and cultural intelligence are two interrelated constructs that play crucial roles in ensuring the success of organizations and individuals in multicultural environments. In today’s increasingly interconnected world, the ability to engage effectively in intercultural interactions is critical, not only for healthcare professionals but also for managers and service providers across diverse sectors. Previous research has consistently shown that individuals equipped with key psychological resources, such as intelligence and adaptability, are more likely to succeed in cross-cultural contexts [23].

Cultural intelligence significantly contributes to the development of cultural competence, as individuals with greater cultural intelligence can better navigate unfamiliar cultural settings, interpret cultural cues, and adjust their behaviors to function effectively in diverse environments. This is reflected in the current study, where cultural intelligence was identified as a significant predictor of cultural competence among nursing students. Notably, the highest mean score for cultural intelligence was found in the cognitive domain. In contrast, the metacognitive domain had the lowest score, suggesting that although students possess cultural knowledge, their reflective awareness of cultural situations and strategy development needs improvement [24]. These findings are consistent with those of the study by Hua et al. [25], which examined the relationships among proactive personality, cultural intelligence, and intercultural adaptation among international students in China. Their results demonstrated that a proactive personality positively correlates with both cultural intelligence and intercultural adaptation. This suggests that personal disposition influences the development of cultural intelligence and, consequently, cultural competence, a notion that resonates with the current study, as demographic factors such as gender, marital status, and field of study also significantly influence cultural intelligence and competence.

Similarly, Iskakova et al. [26] investigated the effects of cultural intelligence and intercultural adaptation on the academic performance of international students in Australia. Her findings indicated a positive relationship between cultural intelligence and intercultural adaptation, aligning with the present study’s demonstration of a significant association between cultural intelligence and cultural competence. However, Iskakova noted a negative relationship between these constructs and academic performance, suggesting potential challenges in balancing cultural adaptation with academic demands — an aspect worth exploring in future studies involving healthcare students. In the aviation service sector, Suthatorn et al. [27] investigated the relationships among cultural intelligence, intercultural communication, and anxiety among Thai airline cabin crew members. Their findings highlighted the role of cultural intelligence in reducing job-related anxiety and improving intercultural communication competence, which in turn enhanced service quality. Although this study involved a different professional context, the implications are relevant to nursing students, who, like cabin crew members, frequently interact with individuals from diverse cultural backgrounds. The positive associations reported in both studies emphasize the practical value of fostering cultural intelligence in service-based professions [27].

In the present study, cultural intelligence emerged as a significant predictor of cultural competence among students in nursing faculties. The highest mean score was observed in the cognitive domain of cultural intelligence, whereas the metacognitive domain had the lowest score. These findings suggest that while students possess cultural knowledge, their reflective awareness of cultural situations and their ability to develop appropriate strategies need further enhancement. These results are consistent with findings from recent international research. For example, Cieślak et al. (2025) explored the association between combined formal and informal educational approaches and cultural intelligence among undergraduate students in nursing faculties. Their results indicated that integrating formal education with informal learning experiences positively contributes to the development of cultural intelligence, ultimately enhancing intercultural interactions in clinical settings [28]. Similarly, Berşe et al. (2025) examined cultural intelligence and migration intentions among nursing and midwifery students in southeastern Turkey. They reported that higher levels of cultural intelligence were associated with a greater intention to migrate, as students with better intercultural skills felt more prepared to work in international environments [29]. In another relevant study, Montayre and Skaria (2025) evaluated the effectiveness of a faculty development program aimed at enhancing the cultural intelligence and intercultural effectiveness of nurse educators. The program significantly improved educators’ intercultural skills, although the authors recommended incorporating real-life case studies and scenarios to further strengthen its impact [30]. Furthermore, Sharma and Hussain (2025) investigated the role of body image and cultural intelligence in the adaptation process of racial minority migrants. Their findings highlighted that greater cultural intelligence helps individuals adjust more effectively to new environments and better manage migration-related challenges [31]. These studies collectively support the conclusion of the current research that cultural intelligence plays a crucial role in shaping cultural competence and facilitating successful intercultural engagement. Therefore, emphasizing the development of cultural intelligence in nursing education is essential for preparing students to provide culturally sensitive care in increasingly diverse healthcare settings.

Additionally, Wang et al. [32] examined cultural competence among Chinese international students in the United States, focusing on the role of cultural intelligence. Their study revealed that the development of cultural competence is influenced by a range of personal, social, and environmental factors, including host community connections, language barriers, and family support. They emphasized the importance of cultural intelligence workshops in promoting adaptation—a recommendation that aligns with the present study’s implications for integrating cultural intelligence training into nursing education programs [32]. Closer to the healthcare setting, Yadollahi et al. [33] conducted a descriptive study assessing cultural competence among nurses in Iran. Using a framework based on six dimensions—cultural flexibility, skill, awareness, desire, care intention, and assessment skill—they reported moderate overall competence, with the highest scores for cultural flexibility and the lowest for cultural desire. This mirrors the findings of the current study, where flexibility also received the highest mean score for cultural competence, whereas cultural desire was the lowest. These findings suggest that while healthcare professionals may possess the adaptability to handle cultural differences, their motivation and willingness to engage in culturally competent care remain areas that need attention. Yadollahi et al. further reported that cultural competence increased with work experience, underscoring the importance of both educational interventions and clinical exposure in cultivating these skills [33].

In the present study, the field of study emerged as a significant predictor with a negative association with CQ. One plausible explanation is that students enrolled in more technically intensive or specialized nursing programs may have fewer opportunities for intercultural exposure and reflective learning than those in broader or patient-facing curricula. Indeed, studies have shown that engaging students in cross-cultural interactions with patients significantly enhances their cultural competence and confidence [34]. Additionally, academic degree level and duration of education have been identified as influential factors in the development of cultural intelligence among nursing professionals. For instance, Atalla et al. reported that nurses with higher educational attainment and greater professional experience scored significantly higher in terms of cultural intelligence [35].

Taken together, the findings of the present study, alongside those of related studies, underscore the complex, multidimensional nature of cultural competence and highlight cultural intelligence as a crucial factor influencing it. This study has several limitations. First, its cross-sectional design does not allow for causal interpretations. Second, the use of self-report questionnaires may be subject to response bias. Finally, the sample was limited to two faculties, which may affect the generalizability of the findings. Future longitudinal and multi-institutional studies are needed to validate and expand upon these results.

## Conclusion

The findings of this study indicate a statistically significant relationship between cultural intelligence and cultural competence. The highest scores were associated with the flexibility dimension, reflecting nursing students’ ability to adapt to individuals from different cultural backgrounds. In contrast, the lowest scores were related to cultural desire, which may suggest the presence of cultural differences and a lack of willingness to understand them. Additionally, metacognitive awareness can be enhanced through education and training. Therefore, by strengthening various dimensions of cultural intelligence and resulting cultural competence, it is possible to improve the quality of healthcare services. The significant relationships between cultural intelligence, demographic variables, and cultural competence identified here suggest a need for targeted educational strategies aimed at enhancing not only students’ cultural knowledge but also their reflective, motivational, and behavioral competencies. Despite Iran’s multicultural society, the national nursing curricula have seldom addressed cultural issues, with limited dedicated courses on cultural competence or transcultural nursing. This gap hampers the development of nurses’ ability to provide culturally sensitive care. To bridge this gap, it is imperative to integrate cultural competence training into nursing education, ensuring that future nurses are equipped to meet the diverse needs of the populations they serve [36, 37]. Additionally, providing faculty development, regular student assessment, and policy support to ensure sustainable implementation across educational settings is suggested. Future research should investigate the longitudinal effects of targeted educational interventions on cultural intelligence and competence among healthcare students and professionals. This would inform evidence-based curriculum planning and help establish best practices in multicultural nursing education.

## Data Availability

The datasets used and analyzed during the current study are available from the corresponding author upon reasonable request.

## References

[1] Osmancevic S, Großschädl F, Stijic M, Lohrmann C. The German version of the cultural competence assessment (CCA-G): cross-cultural adaptation and validation study in Austrian acute care settings. BMC Nurs. 2022;21(1):77. 35365142 10.1186/s12912-022-00854-w PMC8973569

[2] Qin Y, Chaimongkol N. Simulation with standardized patients designed as interventions to develop nursing students’ cultural competence: a systematic review. J Transcult Nurs. 2021;32(6):778–89. 34120529 10.1177/10436596211023968

[3] Sung S, Park H-A. Effect of a mobile app-based cultural competence training program for nurses: A pre-and posttest design. Nurse Educ Today. 2021;99:104795. 33621852 10.1016/j.nedt.2021.104795

[4] Leininger MM, McFarland MR. Culture care diversity and universality: A worldwide nursing theory. Jones & Bartlett Learning. 2006.

[5] Campinha-Bacote J. The process of cultural competence in the delivery of healthcare services: a model of care. J Transcult Nurs. 2002;13(3):181–4. 12113146 10.1177/10459602013003003

[6] Reynolds CL, Leininger M. Madeleine Leininger: Cultural care diversity and universality theory. 1993.

[7] Saleh A. Ethnic identity and the state in Iran. Springer. 2013.

[8] Farzanfar R. Ethnic groups and the state: azaris, Kurds and Baluch of Iran. Massachusetts Institute of Technology. 1992.

[9] Ghanbari H, Rahimian M. Persian Language dominance and the loss of minority Languages in Iran. Open J Social Sci. 2020;8(11):8–18.

[10] Anonby E, Taheri-Ardali M, Hayes A. The atlas of the languages of Iran (ALI): A research overview. Iran Stud. 2019;52(1–2):199–230.

[11] Fadaeinia MM, Miri S, Azizzadeh Forouzi M, Roy C, Farokhzadian J. Improving cultural competence and self-efficacy among postgraduate nursing students: results of an online cultural care training program. J Transcult Nurs. 2022;33(5):642–51. 35684956 10.1177/10436596221101925

[12] Almutairi AF, Dahinten VS. Construct validity of almutairi’s critical cultural competence scale. West J Nurs Res. 2017;39(6):784–802. 27353640 10.1177/0193945916656616

[13] Almutairi AF, Adlan AA, Nasim M. Perceptions of the critical cultural competence of registered nurses in Canada. BMC Nurs. 2017;16:1–9. 28824334 10.1186/s12912-017-0242-2 PMC5558749

[14] Earley PC, Ang S. Cultural intelligence: Individual interactions across cultures. 2003.

[15] Steyn R, Solomon A. Cultural intelligence: concepts and definition statements. South Afr J Bus Manage. 2017;48(2):67–74.

[16] Thomas DC, Elron E, Stahl G, Ekelund BZ, Ravlin EC, Cerdin J-L, et al. Cultural intelligence: domain and assessment. Int J Cross Cult Manage. 2008;8(2):123–43.

[17] Ahmady GA, Nikooravesh A, Mehrpour M. Effect of organizational culture on knowledge management based on Denison model. Procedia-Social Behav Sci. 2016;230:387–95.

[18] Ang S, Van Dyne L, Koh C. Personality correlates of the four-factor model of cultural intelligence. Group Organ Manage. 2006;31(1):100–23.

[19] Karroubi M, Hadinejad A, Taghavian Noghan SA. Studying the relationship between cultural intelligence and emotional intelligence among outgoing tour leaders in Tehran. J Tourism Plann Dev. 2016;4(15):24–41.

[20] Almutairi AF, Dahinten VS. Factor structure of almutairi’s critical cultural competence scale. Administrative Sci. 2017;7(2):13.

[21] Ang S, Van Dyne L, Koh C, Ng KY, Templer KJ, Tay C, et al. Cultural intelligence: its measurement and effects on cultural judgment and decision making, cultural adaptation and task performance. Manage Organ Rev. 2007;3(3):335–71.

[22] Basińska MA, Drozdowska M. Emotional intelligence as an indicator of satisfaction with life of patients with psoriasis. Adv Dermatology Allergology/Postępy Dermatologii I Alergologii. 2013;30(6):365–72. 10.5114/pdia.2013.39435 PMC3907901 24493999

[23] Cotter KC, Reichard RJ. Developing cultural competence through engagement in cross-cultural interactions. Advances in global leadership. Emerald Publishing Limited. 2019. pp. 49–78.

[24] Rafieyan V. Relationship between cultural intelligence and translation of culture-bound texts. J Appl Linguistics Lang Res. 2016;3(3):173–84.

[25] Hu S, Liu H, Zhang S, Wang G. Proactive personality and cross-cultural adjustment: roles of social media usage and cultural intelligence. Int J Intercultural Relations. 2020;74:42–57.

[26] Iskhakova M. Does cross-cultural competence matter when going global: cultural intelligence and its impact on performance of international students in Australia. J Intercultural Communication Res. 2018;47(2):121–40.

[27] Suthatorn P, Charoensukmongkol P. Cultural intelligence and airline cabin crews members’ anxiety: the mediating roles of intercultural communication competence and service attentiveness. J Hum Resour Hospitality Tourism. 2018;17(4):423–44.

[28] Cieślak I, Jaworski M, Panczyk M, Barzykowski K, Majda A, Theofanidis D, et al. Exploring the association between combined formal and informal educational approaches and cultural intelligence among undergraduate nursing students: A cross-sectional study. Nurse Educ Today. 2025;146:106537. 39674054 10.1016/j.nedt.2024.106537

[29] Berşe S, Tosun B, Dirgar E, Yava A. Cultural intelligence and migration intentions among nursing and midwifery students in southeastern region of turkey: A correlational study. J Adv Nurs. 2025;81(4):1914–23. 39304320 10.1111/jan.16463 PMC11896834

[30] Montayre J, Skaria R. A study to identify the effectiveness of a faculty development programme in enhancing the cultural intelligence and intercultural effectiveness of nurse educators. Teach Learn Nurs. 2025;20(2):e509–16.

[31] Sharma N, Hussain D, Navigating, Migration. Role of body image and cultural intelligence in racial minority adaptation. Migration and Development. 2025:21632324241310940.

[32] Wang KT, Heppner PP, Wang L, Zhu F. Cultural intelligence trajectories in new international students: implications for the development of cross-cultural competence. Int Perspect Psychol. 2015;4(1):51–65.

[33] Yadollahi S, Ebadi A, MolaviNejad S, Asadizaker M, Saki Malehi A. Evaluation of cultural competency in clinical nurses: A descriptive study. Avicenna J Nurs Midwifery Care. 2020;28(3):163–70.

[34] Soleimani M, Yarahmadi S. Cultural competence in critical care nurses and its relationships with empathy, job conflict, and work engagement: a cross-sectional descriptive study. BMC Nurs. 2023;22(1):113. 37046274 10.1186/s12912-023-01285-x PMC10091659

[35] Atalla ADG, Elseesy NA. Cultural intelligence and professional competencies among nurses: A cross-sectional study. Alexandria Sci Nurs J. 2023;25(1):151–64.

[36] Amiri R, Heydari A. Nurses’ experiences of caring for patients with different cultures in mashhad, Iran. Iran J Nurs Midwifery Res. 2017;22(3):232–6. 28706549 10.4103/1735-9066.208156 PMC5494954

[37] Asgari P, Navab E, Bahramnezhad F. Comparative study of nursing curriculum in nursing faculties of canada, turkey, and Iran according to SPICES model. J Educ Health Promotion. 2019;8(1):120. 10.4103/jehp.jehp_392_18 PMC6615139 31334272

